# Benefits, barriers and recommendations for youth engagement in health research: combining evidence-based and youth perspectives

**DOI:** 10.1186/s40900-024-00607-w

**Published:** 2024-09-02

**Authors:** Katherine Bailey, Brooke Allemang, Ashley Vandermorris, Sarah Munce, Kristin Cleverley, Cassandra Chisholm, Eva Cohen, Cedar Davidson, Asil El Galad, Dahlia Leibovich, Trinity Lowthian, Jeanna Pillainayagam, Harshini Ramesh, Anna Samson, Vjura Senthilnathan, Paul Siska, Madison Snider, Alene Toulany

**Affiliations:** 1 Temerty Faculty of Medicine, University of Toronto, Toronto, ON Canada; 2 Institute of Health Policy, Management and Evaluation, University of Toronto, Toronto, ON Canada; 3 Child Health Evaluative Sciences, SickKids Research Institute, Toronto, ON Canada; 4 Department of Pediatrics, Faculty of Medicine, University of Toronto, Toronto, ON Canada; 5 Division of Adolescent Medicine, The Hospital for Sick Children, 555 University Ave, Toronto, ON M5G 1X8 Canada; 6 KITE, Toronto Rehabilitation Institute, University Health Network, Toronto, ON Canada; 7 Rehabilitation Sciences Institute, University of Toronto, Toronto, ON Canada; 8 Department of Occupational Science and Occupational Therapy, University of Toronto, Toronto, ON Canada; 9 Lawrence S. Bloomberg School of Nursing, University of Toronto, Toronto, ON Canada; 10 Margaret and Wallace McCain Centre for Child, Youth & Family Mental Health, Centre for Addiction and Mental Health, Toronto, ON Canada; 11 Faculty of Medicine and Dentistry, University of Alberta, Edmonton, AB Canada; 12 Department of Psychology and Neuroscience, Dalhousie University, Halifax, NS Canada; 13 Neurosciences and Mental Health, SickKids Research Institute, Toronto, ON Canada; 14 Michael De Groote School of Medicine, McMaster University, Hamilton, ON Canada; 15 McGill University, Montreal, QC Canada; 16 Department of Health Sciences, University of Ottawa, Ottawa, ON Canada; 17 McMaster University, Hamilton, ON Canada; 18 Collaborator, Toronto, ON Canada; 19 Patient Partner, Canadian Arthritis Patient Alliance, Toronto, ON Canada

**Keywords:** Youth engagement, Patient-oriented research, Narrative review

## Abstract

**Background:**

Youth engagement refers to the collaboration between researchers and youth to produce research. Youth engagement in health research has been shown to inform effective interventions aimed at improving health outcomes. However, limited evidence has identified promising practices to meaningfully engage youth. This synthesis aims to describe youth engagement approaches, frameworks, and barriers, as well as provide both evidence-based and youth-generated recommendations for meaningful engagement.

**Main body:**

This review occurred in two stages: 1) a narrative review of existing literature on youth engagement and 2) a Youth Advisory Council (YAC) to review and supplement findings with their perspectives, experiences, and recommendations. The terms ‘youth engagement’ and ‘health research’ were searched in Google Scholar, PubMed, Web of Science, Scopus, and PsycINFO. Articles and non-peer reviewed research works related to youth engagement in health research were included, reviewed, and summarized. The YAC met with research team members and in separate youth-only forums to complement the narrative review with their perspectives. Types of youth engagement include participation as research participants, advisors, partners, and co-investigators. Barriers to youth engagement were organized into youth- (e.g., time commitments), researcher- (e.g., attitudes towards youth engagement), organizational- (e.g., inadequate infrastructure to support youth engagement), and system-level (e.g., systemic discrimination and exclusion from research). To enhance youth engagement, recommendations focus on preparing and supporting youth by offering flexible communication approaches, mentorship opportunities, diverse and inclusive recruitment, and ensuring youth understand the commitment and benefits involved.

**Conclusions:**

To harness the potential of youth engagement, researchers need to establish an inclusive and enabling environment that fosters collaboration, trust, and valuable contributions from youth. Future research endeavors should prioritize investigating the dynamics of power-sharing between researchers and youth, assessing the impact of youth engagement on young participants, and youth-specific evaluation frameworks.

**Supplementary Information:**

The online version contains supplementary material available at 10.1186/s40900-024-00607-w.

## Abstract (plain language summary)

Engaging and partnering with youth in research related to healthcare is important, but often not done well. As researchers, we recognize that youth perspectives are needed to make sure we are asking the right questions, using appropriate research methods, and interpreting the results correctly. We searched the literature to identify challenges researchers have faced engaging youth in health research, as well as strategies to partner with youth in a meaningful way. We worked closely with 11 youth from across Canada with experience in healthcare, who formed a Youth Advisory Council. The youth advisors reviewed the literature we found and discussed how it fit with their own experiences and perspectives through group meetings with the research team. Youth advisors divided into four groups to co-author parts of this paper, including identifying the importance, benefits, and challenges of engaging in research and providing reflections on their positive and negative previous experiences as youth advisors. This paper provides an overview of recommendations for researchers to engage with youth in a meaningful way, including how they communicate and meet with youth, recognize their contributions, and implement feedback to improve the experiences of youth partners.

**Supplementary Information:**

The online version contains supplementary material available at 10.1186/s40900-024-00607-w.

## Introduction

Patient engagement in health research is essential to improving the relevance, processes, and impact of their findings [1–3]. Defined as the collaboration between researchers and those with lived experience in planning and conducting research, interpreting findings, and informing knowledge translation activities [1], patient engagement in research has been shown to produce and disseminate findings that are more applicable and comprehensible for patients, their families, and the greater community [3–7]. Youth engagement refers specifically to the involvement of youth populations in the research process, with youth often being defined as young people between the ages of 15 to 24 years old [8–11]. Youth, particularly those with chronic physical health (e.g., cystic fibrosis, congenital heart disease, diabetes), mental health (e.g., anxiety, depression), and neurodevelopmental conditions (e.g., cerebral palsy), face unique challenges in engaging with the healthcare system compared to adult populations. These include navigating healthcare transitions, developing relationships with multiple care providers, learning to advocate for themselves, and assuming greater responsibility for their healthcare as they grow and mature [12, 13]. Existing research has shown that engaging youth in research leads to more effective and impactful interventions, policies, and healthcare services aimed at supporting health outcomes of young people, informed by the priorities and experiences of youth themselves [14–19]. Several nationally representative child health organizations and leaders have identified youth engagement as a priority area in youth health, highlighting the urgent imperative to include their voices in health research and public policy decisions [20]. Despite the evidence suggesting that youth are eager and capable of being engaged, there is limited evidence on the unique considerations needed to meaningfully involve youth in health research given their distinct developmental stage [8, 10, 19, 21–29]. These considerations include an emphasis on peer connections, mentorship, flexibility given competing priorities, and the use of technology to allow for broad participation [30, 31]. In collaboration with a Youth Advisory Council (YAC), this review aims to:

1. Outline key types of youth engagement identified in the literature (Aim 1);
2. Review existing youth engagement frameworks identified in the literature (Aim 2);
3. Explore barriers to youth engagement identified in the literature and from YAC member perspectives (Aim 3);
4. Summarize recommendations for engaging youth in research identified in the literature and from YAC member perspectives (Aim 4).

The YAC identified a secondary aim, which was to:

5) Describe the benefits and impact of youth engagement from YAC member perspectives (Aim 5).

## Methods

This project was comprised of two phases. First, the research team conducted a narrative review of the literature. Next, a project-specific YAC was established to review the literature findings and integrate the essential insights and perspectives of youth into the project. The methods pertaining to each phase are elaborated upon below. Our Research Ethics Board did not require a formal review of this project as it did not involve research participants.

### Phase 1: Narrative Review

A narrative review was conducted to explore existing research on engaging youth in health research. Narrative review methodology is often employed to broadly describe the current state of the literature and provide insights for future research [32]. This review method was chosen to establish a broad understanding of the youth engagement literature and provide recommendations for researchers seeking to gain an overview of strategies for meaningful engagement. Narrative reviews also provide flexibility in terms of methodology (often based on the subjectivity of the research team) [33] and are less formal than other types of knowledge syntheses (e.g., systematic reviews) [34, 35]. This review methodology allowed the research team to prioritize and integrate the perspectives of youth into the synthesis of information. Aims 1 to 4 were addressed in Phase 1. Aim 5 was not initially identified as an objective by the research team, and was therefore not included in the review of the literature. Upon establishment of the YAC, youth advisors deemed personal reflections on the benefits and impact of youth engagement from their perspectives critical to the manuscript.

#### Inclusion and Exclusion Criteria

Articles included in this narrative review met the following primary inclusion criteria: 1) published in English language, 2) published prior to April 2023, 3) focused on youth engagement in health research, and 4) described key types of youth engagement strategies (Aim 1), youth engagement frameworks (Aim 2), barriers to youth engagement (Aim 3), or recommendations for youth engagement (Aim 4). For the purposes of this review, ‘youth’ was defined as individuals between the ages of 15 to 24 years old, which is consistent with the definition provided by the United Nations [11], and ‘youth engagement’ was defined as the involvement of young people within this age range in research processes. This population was chosen for the focus of this review as the needs of youth are often distinct from children and adults due to their unique developmental stage (e.g., navigating healthcare transitions, increasing autonomy, etc.) [12, 13]. Articles from any geographic location were included. Grey literature, websites, and non-peer reviewed research works (e.g., conference abstracts, theses) were also included using the same criteria as above.

#### Search Strategy and Synthesis

The search terms ‘youth engagement’ and ‘health research’ were searched in Google Scholar, PubMed, Web of Science, Scopus, and PsycInfo. Articles were hand-searched by members of the research team and selected according to the inclusion criteria above. Reference lists of relevant articles were also scanned. While other knowledge syntheses (e.g., systematic or scoping reviews) review all works identified by the literature search, narrative reviews do not aim to be inclusive of all literature available on a given topic [36]. As such, our review of the literature was concluded once we felt that sufficiency was achieved, which was characterized by reviewing works that yielded recurrent concepts. Additionally, the literature was reviewed iteratively following feedback from youth advisors who critically reviewed the narrative review manuscript. Some aspects of the manuscript were deemed critical to expand upon by youth advisors, and literature was reviewed again accordingly.

Relevant peer-reviewed and non-peer reviewed literature was organized and summarized descriptively according to study aims 1 to 4. Barriers to youth engagement were organized into individual-, organizational-, and systems-level. Recommendations for youth engagement were organized into common overarching themes.

### Phase 2: Collaboration with Youth Advisory Council

**Fig. 1:**
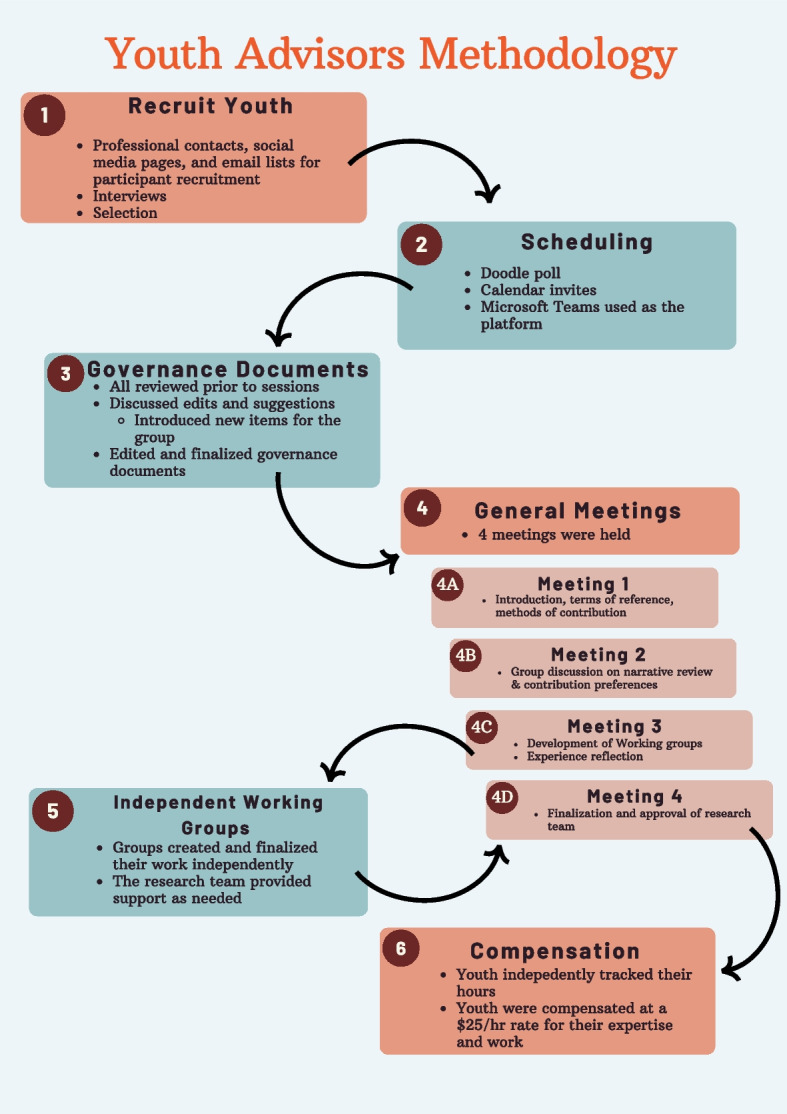
Methodology used to engage the Youth Advisory Council in the co-development of this article. Figure developed by the Youth Advisory Council

The research team identified the criticality of collaborating with youth themselves in the review, formatting, and presentation of findings from the narrative review. As the review was being conducted and written, the research team began recruiting a group of youth advisors to contribute their perspectives, experiences, and recommendations for the manuscript. The development and procedural aspects of the YAC as they relate to the review are described below and in Fig. 1. The operation of the YAC was guided by the McCain Model of Youth Engagement [31] and the Canadian Institutes of Health Research’s (CIHR) Patient Engagement Framework [1]. These frameworks, which prioritize reciprocity, respect, mutual learning, flexibility, and mentorship, supported the use of youth-driven and adaptable engagement strategies throughout the project [1, 31]. Specifically, the research team employed engagement practices including co-building of a terms of reference document, inviting YAC members to co-chair meetings to foster mutual learning, and offering YAC members a menu of options for contribution, that aligned with the principles outlined in these models [1, 31]. Aims 3 (i.e., identifying barriers to youth engagement) and 4 (i.e., summarizing recommendations for youth engagement) were expanded upon by the YAC in Phase 2. As described above, Aim 5 (i.e., benefits and impact of engagement on youth themselves) was deemed crucial by members of the YAC and was exclusively addressed in Phase 2 of this project. It should be noted that while the YAC specifically contributed reflections to Aims 3–5, each member critically reviewed the manuscript and offered feedback as co-authors.

#### Recruitment of Youth Advisory Council Members

Recruitment for the YAC began in June 2023 through distribution of a recruitment poster via professional contacts (e.g., researchers conducting youth-engaged research, youth advisory council facilitators), social media pages, and email lists (e.g., patient-oriented research listservs, youth advisory council lists). Eligible youth advisors were Canadian youth between the ages of 15–24 years with an expressed interest in youth engagement in health research. Youth applicants completed a Google Form to describe their motivations to become involved and past experience, if applicable. To ensure a diverse range of perspectives, we considered age, sex/gender, race and ethnicity, geographic location, and a range of previous experiences with research (from limited to extensive) in our recruitment process. The research team received interest from 55 individuals, of which 17 were invited to complete a 30-min virtual interview co-led by a researcher and a youth research partner. Eleven youth were selected to join the YAC, and all accepted the team’s invitation to participate. The youth invited to compose the YAC predominantly had previous experience with health care, including as a patient, advocate, youth advisor, research participant, or research assistant. Having and/or disclosing a diagnosis of a chronic health condition was not a criterion for participation in the YAC. A collective discussion was held with youth advisors and it was determined that members preferred not to share their demographic information, though there was representation of members with varying ages, ethnicities, years of experience with engagement, and from different provinces. The research team consisted of female-identified researchers, clinicians, and trainees across interdisciplinary professional backgrounds (e.g., medicine, nursing, social work) with experience engaging youth in research and/or clinical care. As many team members do not have previous youth lived experiences in research and/or clinical care, we were committed to closely collaborating and amplifying youth voices in our research, recognizing that our work, interpretations, and applications to the broader community were limited by our non-experiential understanding of youth engagement in research. The composition of the research team and YAC allowed for critical reflection on the roles of positionality, intersectionality, power, and privilege within youth engagement. The team engaged in reflexive discussions about the importance of prioritizing equity and addressing discrimination in engagement, especially for youth with marginalized identities.

#### Scheduling and Meetings

In July 2023, a Doodle Poll link was sent out to all youth advisors to find three meeting times that could accommodate the majority of the youth advisors and research team. Subsequently, Microsoft Teams invites were sent via email, and meetings were recorded and transcribed for notetaking purposes.

Prior to each meeting, a meeting agenda and documents were sent for review. Meetings lasted between 1.5 and 2 h and were recorded for those who could not attend. Both the recording and the minutes were collated following each meeting and made available to all youth advisors. Prior to the first meeting, a draft terms of reference document (ToR) was distributed to all youth advisors for review. The ToR contained the purpose and expectations of youth contributing to the project. A preliminary draft of the narrative review was provided to each youth advisor for their consideration both in advance of and during the meetings. Throughout the meetings, a range of communication methods, including Jamboards, chat messaging, and online verbal discussions, were employed to enable youth to exchange ideas and actively facilitate discussions.

During the initial meeting, youth advisors were provided with guidelines aimed at creating a secure environment using a digital interactive whiteboard on Google Jamboard. To maintain confidentiality and facilitate continuous improvement, the youth advisors proposed and subsequently implemented an anonymous feedback form, accessible for youth to complete at their discretion. Subsequently, the youth advisors engaged in a collaborative ideation session to conceptualize their contributions to the synthesis. It was decided that a Slack channel would serve as the primary platform for communication among the youth advisors.

In the second meeting, the council deliberated on the ToR initially formulated by the research team, with the ToR subsequently revised to incorporate the feedback and insights provided by the youth advisors. Additions to the ToR from YAC members included greater options for compensation, strategies for addressing microaggressions, more clarity regarding YAC tasks, roles, and responsibilities, and rationale for selecting 11 advisors for the group. Following this, the group engaged in a comprehensive discussion centered on their reflections concerning the draft of the narrative review. This dialogue highlighted the identified gaps and obstacles associated with involving youth in research from YAC members’ perspectives, proposed recommendations for future research endeavors, and stressed the importance of integrating youth voices into the research process.

In the third meeting, the focus shifted towards the establishment of more focused working groups. These smaller working groups were structured to address specific aspects, including 1) the rationale behind the research (the “why”), 2) reflections on past experiences with youth engagement, 3) methodologies for engaging youth in the context of this review, and 4) formulating recommendations for future research endeavors. Youth advisors were invited to complete a form to rank their areas of interest in these four areas. Based on their ranked responses, working groups were formed and considered the alignment between youth advisor’s preferred method of contribution (e.g., developing visuals, writing a personal reflection, contributing to a table) and the specific topic of the working group.

During the fourth meeting, which was co-chaired by a research team member and a youth advisor (TL) who volunteered for this role, youth advisors and members of the research team reviewed written materials from each working group, discussed each section of the paper, and reached consensus on how the sections would be presented within the article. It was determined that youth advisor work would be combined with the existing narrative review and showcased using textboxes, figures, and tables.

#### Independent Working Groups

All youth advisors worked in four designated working groups over a 3-week period. Youth advisors communicated via Slack channels, email or personal messaging, with the research team available for support and guidance, as needed. Guidelines for authorship, methods of contributing to each section of the paper (e.g., brainstorming, making point form notes, developing figures), and suggestions on length/format were discussed at YAC meetings. Youth advisors were also provided with a series of resources on a collaborative drive to support their contributions to the review, including a youth-friendly guide to academic writing and examples of reports/journal articles co-authored by youth. All groups worked independently and provided finalized drafts to the research team prior to the fourth meeting.

#### Compensation

All youth advisors were compensated $25 per hour at the end of their involvement. All youth advisors tracked their hours with a maximum of 20 h. Youth advisors were able to track meetings, self-directed work, and all time dedicated to the project outside of meetings.

## Results

A total of 65 articles were included, of which 56 were peer-reviewed and 9 were non-peer reviewed. Of the peer-reviewed articles, 14 were qualitative studies, 12 case studies, 7 mixed-methods, 6 commentaries, 2 curriculum development studies, and 2 randomized controlled trials. Additionally, 13 syntheses were included (*n* = 7 unstructured literature reviews, *n* = 3 scoping reviews, *n* = 2 systematic reviews, *n* = 1 scoping review protocol). Of the non-peer reviewed studies, 4 were websites and 5 were reports. A table is available in Appendix A displaying included article citations, categorization of peer-reviewed versus non-peer reviewed works, and study methods used.

In this section of the article, results pertaining to each of the five aims are presented. Aims 1 to 4 were addressed in Phase 1 of this project to outline types, frameworks, and barriers to youth engagement and summarize the literature’s recommendations on how to meaningfully engage youth. Aims 3 and 4 were addressed in collaboration with youth advisors in Phase 2 to highlight the benefits and barriers of youth engagement and recommendations from the perspectives of the youth advisors on meaningful youth engagement. Aim 5 was identified as a priority for youth advisors and their reflections are provided on the benefits and impact of engagement on youth themselves.

### Aim 1: Key Types of Youth Engagement

There are several approaches to youth engagement in health research, which are based on the aim(s) of a given project, resources available, and preferences of youth themselves (shown in Table 1) [37]. Youth may be involved as *research participants*, such as completing a survey or participating in a focus group [24, 31, 38–40]. Youth may also take on *advisory or consultation roles*, where they provide input on the research scope, recruitment strategies, and methods, as well as reviews analyses, results, and/or manuscripts, from which the researcher may decide if or how to implement their suggestions (e.g., advisory councils) [24, 38–41]. Youth may assume *co-production roles*, which actively involves youth in the development of research objectives and design, funding proposals, study informational materials, recruitment of participants, data collection instruments, co-facilitating focus groups/interviews, analysis of data, presentations, manuscripts, and knowledge translation activities [10, 24, 41]. This may also be referred to as *partnership*, which involves active collaboration of youth with researchers to support and/or lead aspects of the project (e.g., collaborate on research methodology, lead certain research activities) [24, 31, 38–40]. Finally, *youth-led research* refers to projects that are entirely led by youth, with or without the support of an adult researcher [24, 31, 38–40].

**Table 1:** Types of youth engagement in health research

**Type of Youth Engagement**	**Key Features**	**Example(s)**
Participation Role	• Youth are involved as research subjects.	• Adult research teams recruit youth participants to complete a study survey or participate in a focus group, such as conducting semi-structured interviews with youth living with neurodevelopmental disabilities to determine their training needs in becoming research partners [42].
Advisory or Consultation Role	• Youth provide feedback on study methodology, results, and manuscripts developed by the adult researcher. • Researchers may decide the extent to which they implement youth feedback.	• Youth advisory groups/councils, steering committees, such as the MICYRN KidsCan Young Person’s Advisory Group, in which youth advisors consulted on the design of patient information and study materials, recruitment websites, and study design [43].
Co-Production or Partnership Role	• Youth co-produce key components of the study methodology alongside the adult researcher. • Youth are actively involved in data collection, data analysis, and knowledge translation activities. • Youth may support the adult research team or lead certain aspects of the project. • Youth in this role are often included as authors on manuscripts and scholarly outputs.	• Bipolar Youth Action Project, in which youth were engaged as peer researchers in all research phases, including 1) grant writing, 2) recruitment of participants, 3) research participants, and 4) data analysis [44].
Youth-Led Research Role	• All aspects of the research project is entirely youth-led. • Adult researchers may or may not be involved to support the youth research team.	• Sexual Health And Reproductive Empowerment (SHARE) Project, in which youth researchers developed and conducted their own projects to related to sexual health issues [45]. • Youth researchers were mentored by adult researchers [45].

A recent systematic review identified youth engagement practices in mental health-specific research, highlighting the most common youth engagement types were advisory roles, where youth were often involved in providing feedback on the research topic, analysis of qualitative data, and dissemination of findings, with less emphasis placed on co-production methods [10]. Authors identified one study which utilized a youth-led participatory action research approach in the mental health research setting, which is a power-equalizing methodology involving collaborative decision-making and viewing youth as experts based on their own lived experience [44, 46–48].

### Aim 2: Frameworks for Youth Engagement

A significant body of literature has proposed various frameworks for supporting patient engagement in research, with research teams more recently developing frameworks specific to youth engagement [49]. For example, the *Youth Engagement in Research Framework*, designed by youth and researchers at the University of Manitoba, identified seven strategies to create a culturally-inclusive research environment for youth to meaningfully contribute to the research process [50]. Strategies included 1) understanding motivations of youth to engage in research, 2) sharing intentions to implement research findings, 3) supporting diverse youth identities in engagement, 4) actively addressing the barriers to youth engagement, 5) reinforcing that engaging in research is a choice, 6) developing trusting relationships through listening and acknowledging contributions, and 7) respecting different forms of knowledge creation, acquisition, and dissemination [51].

Youth engagement has also been achieved through health research *communities of practice*, a framework aimed at promoting a space for youth to develop identity, build capacity for youth to develop research, communication, and advocacy skills, lead projects, and develop relationships with the research team [52–54]. A Canadian research team developed *IN•GAUGE®,* a health research community of practice which aims to promote collaboration between youth, families, researchers, and policy makers and support the development of strategies to improve child and family health [51, 52]. This program uses Youth and Family Advisory Councils, a group of youth and family members who contribute to the direction of the project and provide input on research methods based on their own lived experiences [51]. This community of practice has built a robust network of youth and family researchers, which helps alleviate some challenges associated with finding youth to support a project.

Researchers at the Centre for Addiction and Mental Health (CAMH) in Toronto, Ontario, Canada have developed the *McCain Model for Youth Engagement,* which is specific to mental health populations [55]. This model is based on *flexibility* (i.e., the youth and research team work together to co-design deliverables/timelines and develop skills that are relevant to the youth’s goals), *mentorship* (i.e., in the development of research skills, incorporating youth strengths into research design), *authentic decision-making* (i.e., avoiding ‘tokenism’, carefully considering and implementing youth feedback), and *reciprocal learning* (i.e., both youth and researchers are ‘teachers’ and ‘learners’). Based on the implementation of the McCain Model, researchers propose that youth engagement should be established when research projects are in the early planning stages, reflect on organizational-level barriers to youth engagement and plan policies and practices around them, and train researchers on the value of engaging youth [55].

A recent commentary made key recommendations for youth engagement in the context of the COVID-19 pandemic [30]. First, authors propose adapting youth engagement strategies to facilitate rapid decision-making, such as utilizing connections with pre-existing youth advisory councils, providing additional compensation, and offering opportunities for online participation. Additionally, they suggest leveraging virtual platforms for youth engagement methods, while ensuring that youth with disabilities or chronic health conditions are offered appropriate accommodations. Finally, subsidies or shared tablets or computers may be offered to youth researchers to ensure virtual platforms are accessible and reduce technological barriers [30].

### Aim 3: Barriers to Engaging Youth in Research

A series of barriers for engaging youth in health research have been identified in the literature through a narrative review. These barriers are grouped into individual, organizational, and systemic factors and are presented below. In Table 2, a summary of these barriers, as outlined in the published literature is presented. Youth advisors were invited to review this list and provide their own expansions, reactions, and additions based on their knowledge and experiences. A key limitation in the exploration of barriers related to youth engagement is that much of the existing literature does not specify what level of youth enagagement was being employed.

**Table 2:** Individual, organizational, and system-level barriers to youth engagement in health research

***From the Literature***	***From Youth Advisors’ Perspectives***
**Individual-Level Barriers: Youth**	
• Mistrust in healthcare system, including healthcare providers and research teams [56]. • Demographic and socioeconomic characteristics (e.g., developmental disability, race and ethnicity, gender, language, health literacy) due to discrimination and negative experiences engaging with the healthcare system and/or research [57, 58]. • Lack of incentive to participate as a youth partner [59]. • Lack of theoretical knowledge about research methods [60]. • Youth attitudes and expectations about the project [61]. • Sustaining youth engagement throughout the duration of the project [61]. • Time commitment to engage in research, other commitments (e.g., employment, education) [61]. • Exacerbation of illness during research time period [44].	• Lack of access to networks or needing “insider knowledge” of research spaces: ◦ Navigating academia depends on knowing who to go to and where youth can find resources to engage in research. ◦ Youth with access to networks or mentors know which researchers or supervisors to connect with to attain a research position, research grants, etc. ◦ Youth without networks or mentors (e.g., first-generation students, new immigrants, parents in other fields, etc.) face a massive learning curve especially in STEM. They are put at a disadvantage, often “behind the curve” and without knowledge of how to become involved in research. • Discriminatory experiences along multiple axes of identities: ◦ Students from traditionally excluded and marginalized backgrounds often encounter discrimination in research spaces. They may not have the tools, resources, or ability to name their experiences with personal and/or institutional discrimination. ◦ This discourages continued participation and engagement in research. It negatively impacts their mental health, making the experience stressful or encouraging them to leave a career in research. They may be further isolated if they cannot find or build a supportive community within these spaces.
**Individual-Level Barriers: Researchers**	
• Pre-existing beliefs, attitudes, and/or biases about youth engagement [24]. • Lack of knowledge about how to engage or communicate with youth [24, 59].	• Competitive attitudes in research: ◦ “Publish-or-perish” attitudes breed an unhealthy and often ableist attitude to work in research. ◦ For example, youth feel forced to take on unpaid labour/“grunt work” at the expense of their wellbeing to get a reference letter from a principal investigator, continue their research position, etc. ◦ Youth researchers might be discouraged from continuing in research because this culture reframes the goal of research from learning and sharpening skills to instead incentivize publishing papers.
**Organizational-Level**	
• Inadequate infrastructure to support youth engagement (e.g., funding, policies, training opportunities) [24]. • Lack of awareness or education for researchers [24, 62]. • Limited access to engagement opportunities and recruitment of youth partners [24]. • Departments/institutions do not recognize or value youth engagement work [24, 62]. • Challenges with Research Ethics Board, with limited best practices for reviewers to evaluate youth engagement practices to ensure methods are ethically acceptable [63, 64].	• Encountering strict hierarchies: ◦ Labs and research spaces construct hierarchies that impose power dynamics (e.g., student < graduate student < PhD candidate < post-doctoral fellow < principal investigator). This creates a space which undervalues youth knowledge or makes it unsafe to step forward and ask for support. • Hiring youth in labs and academia: ◦ An emphasis on credentials and professional experiences (e.g. referrals, resumes, etc.) places youth new to academia or without networks/resources at a disadvantage. How much experience in a lab can researchers expect from a first-year university student hoping to gain experience and learn? • Strict research timelines: ◦ Research is conducted on a per grant, granting agency, or lab project plan timeline but is not flexible for youth circumstances, who are balancing school, work, and/or life. • Equity, diversity, and inclusion (EDI) resources are often overwhelmed or performative: ◦ Having a committee in a department does not translate to youth who are marginalized to navigate academic spaces. ◦ Often, the different resources for EDI-support are disparate, lack connectivity or do not offer support on a sustained basis. • Encountering a culture of individual learning without providing appropriate support: ◦ Many academic research spaces practice a culture of “teach-yourself”. ◦ While independent learning is crucial for building confidence and granting agency over projects, without proper resources and mentorship, this culture leaves young people without the necessary support system to learn the skills they need to be successful in research.
**System-Level**	
• Systemic discrimination and exclusion from research (e.g., Black, Indigenous, and 2SLGBTQIA + individuals) [65–68]. • Stigma of mental health, developmental disability, and/or youth capabilities [69–72]. • Lack of system-wide incentives to involve youth in research, including mandates in academic journals and grant competitions [73]. • Power dynamics between adult researchers and youth [74–77].	• Policy-makers must critically reflect on whose voices are trusted in research spaces and how some voices are silenced because they do not fit a Western worldview around research.

#### Individual-Level Barriers: Youth-Specific

Many youth may be discouraged from engaging in research due to their own negative lived experiences with the healthcare system. For example, youth may be distrustful of adult clinicians and researchers, particularly those who may have had traumatic medical experiences (e.g., lengthy hospital/intensive care unit admissions, surgeries, invasive treatments), complex and chronic healthcare conditions, or marginalized identities [56]. While understanding these perspectives and experiences is crucial to improve health service structures and delivery, they may not be captured without carefully considering and applying appropriate youth engagement methods. Similarly, those with negative previous experiences with youth engagement may feel tokenized or patronized, particularly if they did not feel authentically valued or listened to by the research team [57, 59].

Youth characteristics may also result in exclusion from youth engagement and/or exacerbate existing barriers to partnering, particularly the presence of physical disabilities, visual/hearing impairments, intellectual disabilities, neurological conditions, mental health conditions, and/or socioeconomic factors [69, 70, 78]. Youth with disabilities may experience mobility impairments preventing them from easily attending research team meetings, may require additional time and supports to complete research tasks, or utilize assistive devices (e.g., communication tools) [69, 70, 78]. Low literacy levels and/or language barriers may also make engagement inaccessible without appropriate accommodations [78].

Furthermore, youth priorities may impact willingness to engage in research. Specifically, youth may not feel valued without formal recognition for their contributions, such as financial compensation, volunteer hours, authorship on manuscripts, or opportunities to present research at academic meetings [59]. They may also not want youth engagement opportunities to infringe on their leisure or personal time, or may be hesitant to engage in projects with long time commitments [61]. A study highlighting experiences with engaging youth with Bipolar Disorder as peer researchers identified that attrition was also affected by illness relapse, as well as difficulties balancing the responsibilities of the research project with post-secondary education and employment commitments [44].

#### Individual-Level Barriers: Adult Researcher-Specific

Research team members may also hold specific beliefs or attitudes towards youth engagement. For example, some researchers may feel anxious about losing control over the research process, may not see youth as experts themselves, or hold biases about the value of youth perspectives [24]. Researchers may also perceive youth engagement as an added layer of complexity, fear that engagement may impact the scientific rigor of the research design, or be concerned that youth engagement may negatively impact the research quality [24, 26, 27, 79–81]. Further, some studies have highlighted that researchers do not feel equipped with the skills or knowledge to engage and communicate with youth, or to design studies using youth engagement principles [24, 62]. Finally, researchers may experience challenges navigating differing priorities between youth partners and members of the research team. For example, researchers may prioritize more traditional markers of research success, including peer-reviewed manuscripts and grant proposals which often require rapid turnaround times, and be concerned that youth engagement may add to the timeline of a project [24, 62].

#### Organizational-Level Barriers

As youth engagement has emerged as a best practice recently, many academic institutions do not yet have the infrastructure or resources to support engagement opportunities [24]. While examples of capacity-building programs for youth co-researchers exist in the participatory action research literature [82], there is a need for further development of training resources to support youth who are engaging in health research [83]. Formal education on youth engagement is often not included in research training programs, despite many granting agencies recently making changes to require and/or promote patient engagement considerations in funding applications [1, 62]. Further, many organizations have not adopted policies to outline best practices for youth engagement, and academic workplace culture also may not yet value youth engagement, resulting in limited willingness to adapt research practices [24, 62]. These factors may exacerbate existing difficulties with securing sufficient time and resources to support relationship-building between youth partners and adult members of the research team, which is a commonly cited challenge with youth engagement [26, 27, 84, 85].

#### System-Level Barriers

Youth with complex health conditions, such as those with developmental disabilities, often experience stigma and exclusion from clinical research [69–72]. Specifically, research teams may inaccurately perceive youth with chronic medical conditions as ‘vulnerable’ or ‘fragile’, thus deeming them unable or incapable to contribute meaningfully or complete study-related tasks [24, 70, 72, 73, 86, 87]. Youth with marginalized identities, including Black, Indigenous, and 2SLGBTQIA+ youth, often experience discrimination within the healthcare system, with several studies suggesting mistrust of research institutions, researchers, and healthcare systems stemming from community experiences of mistreatment in research as the most significant barrier to participating in clinical research [65–68]. Furthermore, youth from racial and ethnic minorities often receive less information and attention from healthcare providers compared to white youth, potentially limiting awareness of the opportunities and/or value in contributing to health services research [68, 88]. Notably, limited literature has considered the impact of other social and structural determinants of health on youth engagement, including income, housing, and geographic location.

Youth may also be apprehensive to share their perspectives, critiques, or suggestions for improvement with adult researchers due to inherent power imbalances [74–77]. Given the differences in power between adults and youth, as well as between patients and clinicians/researchers, youth engagement may involve researchers dominating the conversation, thus preventing equal contribution and collaboration. Ultimately, these dynamics have the potential to produce harmful cultures or practices for youth entering research environments, especially among youth from marginalized groups. These barriers and possible outcomes resulting from these power imbalances are elaborated on in Table 2.

Finally, researchers themselves may face barriers as many major funding agencies have yet to prioritize or incorporate youth engagement in their strategy, resulting in limited funding opportunities to support this type of engagement work or a lack of dedicated time and resources for researchers to build relationships with youth [73]. Of note, the CIHR has developed a Strategy for Patient-Oriented Research, and requires grant proposals in certain funding streams to utilize patient engagement methods [1]. However, this is not yet universally implemented across funding agencies and does not guide engagement with youth specifically. Additionally, funding agencies often have strict eligibility and assessment criteria, including level of education and evidence of prior research and scholarly outputs, which may inherently exclude youth researchers from participating in funding applications. Finally, granting agencies have funding deadlines which may not accommodate the flexibility needed to build meaningful relationships with youth partners.

Further, while some academic journals have incorporated mandatory reporting on stakeholder and patient involvement in the research design, this is not a standard of practice, and many of these journals are engagement-focused [55, 62, 89]. Finally, there is a lack of consensus around how to report on engagement practice and outcomes of engagement across studies, which contributes to inconsistencies in what constitutes meaningful and effective engagement. While tools are emerging to enhance transparency in reporting engagement, including the Guidance for Reporting Involvement of Patients and the Public (GRIPP), no tools exist for youth engagement specifically [90, 91]. Barriers to engaging youth in health research from both the literature and the perspectives of the youth advisors involved in this project are summarized in Table 2.

### Aim 4: Facilitators and Recommendations for Youth Engagement

Many studies have highlighted recommendations to improve the implementation of youth engagement across research contexts. Canada’s Youth Policy was created in 2020 to develop a greater understanding of the experiences and perspectives of youth living in Canada [92]. As part of this, funding opportunities through Canada’s major funding body for health research (CIHR) have begun to focus on providing meaningful opportunities to empower youth in research such as the Healthy Youth Initiative [93]. Our study findings are in line with these newly implemented policies as they lay the foundation for researchers on how to meaningfully engage youth in health research. In the following section, current strategies, strengths, and facilitators in the health sector that can support youth engagement are outlined, along with areas for improvement. As in Table 2, these recommendations were reviewed and expanded upon by the YAC in Table 3.

**Table 3:** Summary of evidence-based and co-created Youth Advisory Council recommendations for youth engagement

	**Recommendations from the Literature**	**Recommendations from Youth Advisors**
***Individual-Level Recommendations***		
**Training Opportunities**	• Formally train youth about the research topic and process [10, 83, 94]. • Offer opportunities for youth to practice their skills prior to initiation of the project [10]. • Gradually reduce support for youth as they begin to build competency [10]. • Use established youth researchers to lead youth training sessions [10]. • Have youth co-design and co-deliver youth engagement training to researchers [42]. • Use multiple modes of delivery for youth engagement training, including videos, online modules, mentorship, and resources [42]. • Offer concrete training opportunities for researchers on how to effectively engage youth [62]. • Train all research staff involved on best practices for communicating and leading youth [10]. • Provide youth with opportunities for growth (e.g., attending workshops, conferences) [78].	• Provide preparatory material in advance containing relevant background information, common language and terms to orient the youth, and allow them to feel better equipped to participate. • Provide leadership opportunities through which youth can lead and co-chair meetings as the project progresses to increase youth agency. • Establish a mentorship program through which experienced youth mentors can regularly check in with their beginner youth mentees to ease nerves and increase confidence.
**Team and Youth Recruitment**	• Develop partnerships with youth at the beginning of the research process [55]. • Use social media or youth-friendly flyers that target community organizations that serve youth and youth advisory councils [78]. • Recruit multiple youth at the beginning of a project to mitigate impact of attrition [10]. • Involve a project champion to lead effective engagement strategies [21].	• Use broad recruitment techniques (virtual or in-person postings) in various spaces and target groups to ensure diversity. • Verbalize value of youth’s connections and experiences that can assist in recruitment. • Ensure youth understand commitment, benefits/compensation for project, and expectations at recruitment stage.
**Budget and Incentives**	• Have a flexible budget in case youth propose adjustments to research protocol [10]. • Formally recognize youth contributions, including volunteer hours, course credits, certificates, reference letters, honorariums or wages, co-authorship [44, 58]. • Consult with youth themselves to decide how to best recognize their contributions [59]. • Offer compensation for travel, if applicable [10]. • Discuss future directions of research project and gauge interest of youth in continuing to partner in future research endeavours, if applicable [42].	• Promote enhancing youth’s networks within the research space. • Introduce youth to research colleagues, increasing opportunities that align with youth participant’s values. • Offer financial compensation for time devoted towards research through cash/e-transfer deposits (most preferable), visa cards or gift cards.
**Research Meetings**	• Provide opportunity for written and verbal participation, as well as virtual opportunities [30]. • Offer multiple ways to engage in research meetings, including providing lay summaries of meeting discussions via email, scheduling additional one-on-one meetings, and team discussions [42, 95, 96]. • Offer blended online and offline engagement formats, including use of pre- and post-meeting work to allow for shorter meetings to be scheduled [97]. • Choose meeting spaces that are accessible, accommodating, and account for potential past traumatic experiences within healthcare settings [78]. • Support youth in facilitating research meetings [55]. • Establish timelines that support their participation in the project and be upfront about time commitment expectations [59]. • Be flexible with meeting hours (i.e., offer weekend and evening meeting times to accommodate school/work schedules) [78]. • Assign concrete responsibilities and tasks at the end of each meeting [44]. • Co-design terms of reference at the first meeting to establish group guidelines and create a safer space [31].	• Include anonymous forms of participation such as Jamboard, Mentimeter, etc. • To create a safe space, allocate time throughout the project for youth to share their thoughts, feedback and experiences, which can be done during meetings or anonymously through feedback forms. • To support youth in facilitating meetings, provide meeting agenda templates and encourage goal/next steps planning. Researchers can also include examples of how previous projects have been conducted. • Provide recordings, meeting minutes and asynchronous participation opportunities for youth that are unable to attend any meetings. • Discuss each team member’s strengths and preferred form of contribution before assigning tasks and responsibilities. Researchers can also ask youth if they want to have dedicated roles such as facilitator, note-taker, etc.
**Communication Approaches**	• Use youth-friendly communication strategies, including limiting jargon, using a polite demeanour, and respecting youth contributions and perspectives [62, 78, 98]. • Reassure youth that their input and contributions are valued [44]. • Utilize different levels of youth participation to balance youth schedules (school, work) and project timelines [31]. • Clearly define roles and responsibilities for youth and adult researchers [98, 99]. • Set mutual expectations for communication approaches between researchers and youth [95]. • Discuss youth goals and values to understand what ‘meaningful engagement’ means to them [100]. • Co-develop research goals grounded in the context of the greater purpose of the research project to enhance motivation (e.g., reinforce how small research activities impact greater study) [44]. • Use relationship building activities to establish rapport [101]. • Ensure accessibility in communication approaches, such as both written and audio-recordings of documents, video recordings of meetings with closed captioning enabled) [42]. • Be transparent about what the expected benefits of the research are and who the findings will likely impact (e.g., some youth may not value increasing knowledge generation, but may be motivated to know the outputs of the research may be expected to impact a certain change) [97]. • Be transparent about how youth feedback will be used, such as using tracked changes to research outputs [97].	• Avoid using tokenistic language when involving youth. For example, instead of valuing participants for being youth, emphasize the value of their contributions. • Recognize youth contributions towards the research project. For example, a summary of the benefits of their ideas could be provided over time, such as *“Your idea on posting flyers in this clinic helped us recruit six participants and develop a connection for future research.”* • Provide an overarching aim or direction of the project to aid in the co-development of goals with youth. • Co-develop research goals or use pre-established goals to give direction but that allow for flexibility. • Engage team-building in youth through the use of icebreakers, introductions, group chats or other ideas that excite youth. • Establish how youth would best like to connect with the team. • Allow flexibility in communication platforms such as: Email, Messenger, WhatsApp, Slack. Youth should be involved in the establishment of preferred method(s) of communication. • Consider developing a “response time” for communication to respect youth commitments and completion of the research project.
**Engaging Youth from Diverse Backgrounds**	• Aim to develop methods of recruitment of youth and researchers who may speak to the diverse population and/or population of study [31, 61, 102]. • Acknowledge and value diverse backgrounds, perspectives, and ideas by openly discussing input [103]. • Book a translator and/or use communication devices if working with youth with language and/or communication barriers (e.g., deaf, hard of hearing) [78]. • Creatively explore individualized methods to engage youth with special needs (e.g., involving caregivers, discuss activity preferences, use non-verbal communication methods) [70, 78]. • Develop group boundaries and expectations to create a safe space for all youth involved [78]. • Consider individualized learning needs of youth partners [78]. • Respect that youth facing social stigma may be apprehensive to share their perspectives [78]. • Adhere to ethical principles and best practices for specific communities, if applicable [31]. • Use a trauma-informed approach and identify when topics or situations trigger personal reactions [78, 101].	• Recognize the diversity of youth, in terms of backgrounds and beliefs, without establishing hierarchies around said beliefs or values. • Recognize and engage in meaningful conversation around how research institutions recreate and reinforce certain power structures or dynamics in traditionally marginalized communities (e.g. extraction of knowledge with no acknowledgement for contributions, harms perpetuated from failing to consent, etc.). • Avoid creating notions of “representatives”. Youth from one community should not be expected to speak on behalf of their entire group and others. Recognize that one youth can have specific experiences but it does not mean they speak on the whole. Focus on broadening engagement with multiple members of similar groups/backgrounds. • Have feedback mechanisms that address youth concerns/lack of safety and accountability mechanisms that ensure these issues are not replicated in the future. • Identify and share external resources to ensure youth remain safe as they engage in spaces.
**Evaluation**	• Use formal, evidence-based evaluation tools iteratively, including quantitative and qualitative data about the engagement process, throughout the research process and continually make amendments/incorporate feedback [31, 104]. • Use formal, evidence-based evaluation tools at the end of the research process using mixed methods (e.g., anonymous feedback tools, semi-structured questions) to understand the overall engagement experience [78]. • Use informal check-ins following each meeting to gain youth feedback, either via anonymous tools, verbal feedback in breakout rooms, email, or other communication platforms [78, 97]. • Evaluate both youth and researchers’ perspectives [31].	• Track changes (e.g. in a research decisions log, transition document, etc.) which can be reviewed at the end or applied in other youth projects. Changes can include feedback on what did and did not work. • Work with youth to define what “success” for a project looks like. Create youth-informed benchmarks (quantitative and/or qualitative) that incorporate youth or community standards of success. • Have “first-responders” able to receive complaints from youth and can act on them. Accountability builds safety and rebuilds trust in institutions.
***Organizational and System-Level Recommendations***		
**Capacity Development**	• Provide researchers with practical steps needed to meaningfully engage youth across the continuum of research [62]. • Advocate for funding agencies, journals, Research Ethics Boards, and academic institutions to include mandates to engage youth and/or offer increased recognition and appreciation [62]. • Develop or support local, regional, or national networks of youth-engaged researchers [62]. • Raise awareness of the importance and benefits of using youth engagement to enhance research findings [62]. • Use creative strategies to promote and centralize engagement opportunities, such as a national repository of engagement opportunities [42, 105]. • Organizations may consider profiling youth and researchers interested in youth engagement to cultivate relationships through social media platforms [42, 106].	• Develop “Youth Research Engagement Coordinator” position at research institutions who can offer training to staff and youth for all stages of projects, advertise opportunities, and assist youth programming. • Dedicate website space for accessible and efficient presentation of youth opportunities. • Create Youth Advisory Boards for projects relating to youth. • Determine how youth can feel empowered in their communities. • Partner with schools to offer credit hours, research internships, funding for self-directed project development and more. • Share opportunities via established youth and professional networks and mentorship groups.

#### Engaging Youth from Structurally Marginalized Populations

Engagement of youth with intersecting marginalized identities, such as Black, Indigenous, or 2SLGBTQIA+ youth, and youth with disabilities, language/communication barriers, immigrants and refugees, experiencing homelessness, or living in foster care, may involve several unique considerations [31]. Research teams should engage both youth and researchers from communities with lived experience to provide insights and support engagement strategies [31]. It is also important to recognize that engaging youth from Indigenous communities may involve a unique approach. Practices adopted by Indigenous-led organizations may exist that focus on youth empowerment that are specific to their communities. For example, the *‘Indigenous Youth Voices Report*’ produced by The Yellowhead Institute at Toronto Metropolitan University in collaboration with the First Nations Child and Family Caring Society outlined requirements for engaging and conducting research with and by Indigenous youth, which included themes such as ensuring research is accessible, uplifting Indigenous youth to co-create research, relationship-building and reciprocity, and using holistic approaches to ensure Two-Spirit, 2SLGBTQ+ youth, and Elders are meaningfully included in research approaches [107]. Further, a recent study showed evidence supporting the use of web-conferencing technology to engage Aboriginal and Torres Strait Islander in Australia through co-facilitation of an Online Yarning Circle, an Indigenous methodology that involves sharing, listening, interpreting, and understanding information in an informal setting [108, 109].

Additionally, teams should partner with researchers who have experience working with youth from these populations. Women’s College Hospital in Toronto, Ontario, Canada has recently developed an innovative and inclusive patient engagement model, called *Equity-Mobilizing Partnerships in Community (EMPaCT)*, designed to highlight the priorities and needs of diverse communities informed by the perspectives of individuals with lived experience [110, 111]. Research teams can consult this service to identify approaches to advance equity and social justice within their projects [110, 111]. Researchers may also consider using the *‘Valuing All Voices Framework’*, which is a trauma-informed, intersectional framework that guides researchers on how to embed a social justice and health equity lens into patient engagement, with the goal of enhancing inclusivity within health research [112]. This framework is based on four core concepts, including *trust* (e.g., focusing on resilience/strength rather than challenges, allowing time to build relationships), *self-awareness* (e.g., practicing honesty, creating safe spaces), *empathy* (e.g., allowing the space to share stories), and *relationship building* (e.g., share experiences, promote ongoing communication, show awareness and sensitivity towards cultural differences) [112].

All research team members engaged in this work should be offered training on best practices for communicating and engaging with specific populations [31]. Appropriate accommodations, such as communication tools, accessibility aids, and financial support for involvement, should be offered consistently to optimize engagement of youth with diverse experiences and perspectives [78]. While not specific to youth engagement, the National Health Service in the United Kingdom has a guidance document which outlines considerations to increase diversity in research participation, including a focus on building trust, conducting research in places familiar to participants, developing accessible recruitment materials, and incorporating peer-led activities [113]. Finally, researchers should adhere to existing ethical standards for specific marginalized communities, such as the CIHR guidelines for conducting research involving Indigenous people [114].

#### Evaluation of Youth Engagement

Robust evaluation of youth engagement strategies is a core component of youth involvement in research and should be used to enhance implementation of principles in research, provide feedback, and ensure researchers are held accountable in upholding best practices [104, 115]. While there are no empirically-tested tools for the evaluation of youth engagement in research, qualitative, quantitative, and mixed methods may be used, including the Youth Engagement Guidebook developed through the CAMH [31], the Public and Patient Engagement Evaluation Tool (PPEET) [116], and the Patient Engagement in Research Scale (PEIRS) [117]. These instruments are co-designed by patients and are used to evaluate the quality of engagement strategies from the perspective of patient partners themselves [117]. It should be noted, however, that empirically-tested tools for measuring youth-adult partnerships more broadly do exist [118–120] and could likely contribute useful information to the measurement of youth engagement in research, specifically. It is also recommended to evaluate the impact of youth engagement from the researchers’ perspectives, which may include reflecting on how valuable the team considered youth partners to be, the extent of youth involvement, and the impact of youth engagement on project outcomes [31]. Alberta Health Services has developed a resource tool kit containing survey instruments to assist research teams with routine evaluation of their collaboration skills [121]. Research teams should carefully evaluate and iteratively modify their engagement strategies to ensure youth are meaningfully involved.

#### Capacity Development

Several independent training programs exist to educate researchers, community stakeholders, patients, youth, and caregivers on engaging patients in health research, including the Patient and Community Engagement in Research (PaCER) program [122], McMaster University Family Engagement in Research (FER) course [123], Patient-Oriented Research Curriculum in Child Health (PORCCH) [124], and Partners in Research (PiR) [125]. Further, a recent study was conducted to develop simulations in collaboration with interdisciplinary stakeholders to train researchers on how to engage youth in childhood disability research [126]. These simulation videos focused on aspects of the research process where challenges may arise based on previous experiences of youth and family advisors [126].

### Aim 5: Youth Advisor Reflections on the Impact of Youth Engagement

While describing the evidence-based benefits of youth engagement in research within the literature was beyond the initial scope of the narrative review, youth advisors deemed it critical to present their experiences regarding their motivations for becoming involved in research and the impact of research opportunities on youth. Two youth advisors reflected on the benefits of youth engagement in research from their own experiences and collectively developed the content displayed in Table 4 in a small working group. The same two advisors considered their prior involvement in research and outlined the impact of engagement on their lives in Table 5. They were invited to share any aspects of their experiences they felt were important to communicate with a broad audience, and selected the format and method of organization of their reflections. These reflections offer unique and valuable insights into the importance of creating opportunities for meaningful and conscientious youth engagement in research using youths’ own language.

**Table 4:** Youth advisors’ reflections on the benefits of youth engagement in research

“Every youth involved in research has a unique set of reasons that motivate them to participate, including the personal benefits to each individual. Youth engagement allows youth to explore areas of interest to help them realize areas of strengths and weaknesses. If youth are never given the opportunity to participate and engage in research, it can be difficult for those who are curious about research to find and apply their passions and interests. Regardless of the type of youth partnership opportunity, when collaborating with a research team, youth gain valuable research experience, including the opportunity to network with professionals. Youth who are interested in pursuing post-secondary education in health sciences might be inclined to develop their research skills and develop a network through their involvement as a youth in research. Starting with engagement in research as a youth is an approach that people use to get further involved with research as an interest or career, making it a significant source of motivation. Many youth are motivated to become involved in health research due to their own experiences with healthcare. As patients, caregivers, and those in proximity to health conditions, youth interact with healthcare in a variety of ways. Many cite negative experiences with healthcare due to ageism, where their voices and experiences aren’t taken seriously due to their young age. These experiences fuel many youth to participate in health research so that they can improve healthcare outcomes by advocating for change. Youth want to use their experiences to influence change so that others do not face the same hardships they faced while navigating the healthcare system. By championing and normalizing youth engagement in research, youth want to make sure their perspectives are embedded in research from the start; closing the gap of missing or inadequate data regarding adolescents and young adults in broader research. Many youth are interested in participating in health research to bring the youth perspective to research, improve existing knowledge, and improve the healthcare system and health outcomes for others. Without youth participation in research, there is little to no research about youth or discussing topics that are prevalent to youth in society. Following the “Nothing about us, without us” slogan used by the Disability Rights Movement, youth from diverse backgrounds are encouraged to get involved in research as research covers a large range of topics relevant to outcomes in youth health [127]. By normalizing and encouraging participation in research, youth of all socioeconomic backgrounds can get involved in research, resulting in representative research data. Youth are interested in health research because they have unique perspectives and opinions based on their own experiences. Youth involved in research are able to rebuild their own trust in the medical system by being able to provide their own opinions and perspectives to the research team and feel heard when the research reflects their perspectives. Many youth are left with a sense of purpose after being involved in research and continue engaging themselves in research, which results in more studies being conducted on youth by youth.” ***Written by members of the Youth Advisory Council***

**Table 5:** Youth advisors reflections on the impact of engagement experiences on youth themselves

“Youth engagement in health research is a necessity. Therefore, considerations of their experiences is a necessity. The previous experiences of youth interacting with healthcare or health research will often impact their behavior going further into adulthood. Research is enriched by the participation of all types of people from all demographics. This means that for researchers who want that enrichment in their work, they need to be thinking about what kinds of experiences youth are having that shape their perspectives about the world around them as well as about health and health research. For example, one of the Youth Advisors for this paper, Madison, had joined a previous research coalition for patients with autoimmune disease. Within that project, the lead researchers routinely reiterated that the project was being led by the young adults, and that final decisions needed to be made amongst themselves, not by the researchers. This encouraged members who maybe did not have experience in this area yet to speak up with their peers rather than continuing to let the ‘real adults’ make the decisions for them. As a result, Madison gained confidence in a research space as well as in a co-leadership role, and felt empowered to join more research projects. Another youth who contributed to this paper, Jeanna, was a member of a youth advisory council for a youth health care organization a few years ago. Part of the council’s role was to advise on and support various research projects within the organization. However, as Jeanna spent more time with the organization, contributing her time and lived experience, she began to notice a pattern. It seemed that for many of the projects that she had been a part of, she would either never hear back about the progress of the project again after her initial contribution, or it seemed like none of the youth’s contributions were actually included in the final project. When Jeanna tried to bring this up to her contacts within the organization, promises were always made to address her concerns, but she would never hear from anyone about the issue again. After a while, this started to take a toll on her. She felt like she was not actually making any meaningful change in the health care system, and was failing to be a good advocate for youth in health/research environments. The experience left her feeling tokenized, defeated, and powerless. These two vastly different experiences are a testament to how important it is to meaningfully and conscientiously engage youth in research. During the process of engaging youth in research, it is important to remember that intent and impact are two different things. Youth who come into these spaces come with an abundance of passion, knowledge, and lived experience. They also come with curiosity and a desire to learn. Following best practices to engage these youth is imperative for helping them to maintain trust within the research process. In turn, researchers will receive much stronger research outcomes.” ***Written by members of the Youth Advisory Council***	

## Conclusions, Limitations & Future Directions

This narrative review provides an overview of the current literature in youth engagement in health research in combination with the perspectives of youth advisors themselves. The research team and YAC collectively identified key types and frameworks for youth engagement, synthesized several barriers and recommendations for implementing youth engagement, and provided critical reflections on the impact and benefits of youth engagement in the youth voice. While many evidence-based frameworks exist to incorporate and evaluate patient engagement in research, gaps remain in the identification of the best practices for youth engagement specifically [49]. Much of the available youth engagement literature has focused on involving youth in mental health research, with limited evidence regarding best practices to engage youth with chronic physical health and neurodevelopmental conditions [10, 21, 24]. Further, a paucity of evidence has highlighted the barriers and best practices to engaging youth with low income, those experiencing homelessness, and rural/remote communities in health research.

### Limitations

This article employed narrative review methodology to provide an overview of existing research in youth engagement in research. A more structured and systematic review and critical appraisal of included literature by multiple independent reviewers was not within the scope of this paper, which may have excluded relevant literature. The information presented in this article may serve as a foundation for a systematic review of the literature on this topic, which our research team endeavours to complete in the future. Additionally, the search was limited to articles published in English, which may have excluded relevant literature, including potential barriers or recommendations specific to non-English speaking youth. Future research should consider a fulsome exploration of youth engagement strategies, barriers, and recommendations published in languages other than English. Demographic information of youth advisors was not collected or presented as part of this article due to YAC member preference. In addition, a previous diagnosis of a chronic health condition and/or lived experience as a patient was not a criterion for inclusion in the YAC. Rather, youth advisors had a diverse set of experiences with health care (e.g., as patients, advocates, previous youth advisors, research assistants, and/or research participants). Furthermore, youth members were self-selected by the research team, and not recruited from established youth organizations with elected representatives. As such, we are unable to determine whether the youth composing the YAC are representative of the target population. Future studies could examine how demographic characteristics and/or prior experiences with engagement influence youths’ perceptions of barriers, enablers, and recommendations for youth engagement.

### Future Directions

To address many of the barriers identified in this review, further work is needed at the organizational- and systems-levels to build policies and programs that support youth engagement in research. As such, youth advisors developed a call to action for researchers and their hopes for the future of youth engagement in research, available in Table 6. Finally, robust studies are needed to develop and validate youth engagement evaluation tools [31].

**Table 6:** Youth Advisory Council call to action for researchers

“In the future we hope to see more youth involved in research for personal and professional purposes. We hope that youth researchers have a voice and are valued. Transforming research spaces to advocate and empower youth requires change from individual to systemic levels. As youth-specific interventions are implemented, as indicated by the youth and research team, it is the Youth Advisory Council’s hope that: • Power is re-shared and re-negotiated, moving away from traditional hierarchies and towards a culture and institutional practices that ground their research in youth and community expertise. • Researchers gain knowledge about allyship with youth and what it looks like to mitigate unhealthy behaviours that arise from power dynamics. • Professionals are dedicated to youth research engagement and reflect on how they can engage youth in their own work. • Youth know where to find research opportunities and are not held back from acting on their ideas due to lack of resources, support, and knowledge.” ***Written by the Youth Advisory Council***	

### Supplementary Information

Supplementary Material 1

## Data Availability

No datasets were generated or analysed during the current study.

## Abbreviations

YAC: Youth Advisory Council
ToR: Terms of reference
