# Supplementary material for: Benefits, barriers and recommendations for youth engagement in health research: combining evidence-based and youth perspectives
Source: Res Involv Engagem. 2024 Sep 2;10:92. doi: 10.1186/s40900-024-00607-w (PMC11370084; doi:10.1186/s40900-024-00607-w)
Supplement: Supplementary file 1 — Supplementary Material 1 [file 40900_2024_607_MOESM1_ESM.pdf]

Appendix A. Included article citations, categorization into peer vs. non-peer reviewed, and methods used.

Author (Year)	Peer vs. Non-Peer Reviewed	Methodology
Wang et al (2023)	Peer-reviewed	Scoping review
Allemang et al (2021)	Peer-reviewed	Commentary
Allsop et al (2010)	Peer-reviewed	Literature review
Anderson et al (2021)	Peer-reviewed	Qualitative study
Bailey et al (2015)	Peer-reviewed	Systematic review
Bennett et al (2022)	Peer-reviewed	Mixed-methods
Beresford (2012)	Peer-reviewed	Case study
Canadian Institutes of Health Research (2023)	Non-peer reviewed	Website
CanChild (2023)	Non-peer reviewed	Website
Catino et al (2019)	Non-peer reviewed	Report
Cavens et al (2002)	Peer-reviewed	Qualitative study
Chan et al (2021)	Peer-reviewed	Mixed-methods
Clavering et al (2010)	Peer-reviewed	Literature review
Darnay et al (2019)	Non-peer reviewed	Report
Delman (2012)	Peer-reviewed	Commentary
Dewa et al (2019)	Peer-reviewed	Qualitative study
Dewa et al (2021)	Peer-reviewed	Case study
Dong et al (2023)	Peer-reviewed	Mixed-methods
Edwards et al (2016)	Peer-reviewed	Qualitative study
Ennals et al (2022)	Peer-reviewed	Qualitative study
Faithfull et al (2019)	Peer-reviewed	Qualitative study

Appendix A. Included article citations, categorization into peer vs. non-peer reviewed, and methods used.

Fayant et al (2020)	Non-peer reviewed	Report
Fløtten et al (2021)	Peer-reviewed	Scoping review
Hawke et al (2017)	Peer-reviewed	Scoping review protocol
Hawke et al (2018)	Peer-reviewed	Commentary
Hawke et al (2020)	Peer-reviewed	Qualitative study
Heffernan et al (2017)	Peer-reviewed	Case study
Henderson et al (2018)	Peer-reviewed	Randomized-control trial
Henderson et al (2022)	Peer-reviewed	Randomized-control trial
Holland et al (2010)	Peer-reviewed	Mixed-methods
Inspiring Healthy Futures (2021)	Non-peer reviewed	Report
Kembhavi et al (2009)	Peer-reviewed	Case study
Kendal et al (2017)	Peer-reviewed	Qualitative study
Kim (2016)	Peer-reviewed	Literature review
Kirk (2007)	Peer-reviewed	Literature review
Krenichyn et al (2007)	Peer-reviewed	Commentary
Lapadat et al (2020)	Peer-reviewed	Mixed-methods
Larsson et al (2018)	Peer-reviewed	Scoping review
Liebenberg (2017)	Peer-reviewed	Letter from the editor
Lincoln et al (2015)	Peer-reviewed	Mixed-methods
Macarthur et al (2021)	Peer-reviewed	Curriculum development
Mawn et al (2015)	Peer-reviewed	Case study
McCabe et al (2023)	Peer-reviewed	Systematic review

Appendix A. Included article citations, categorization into peer vs. non-peer reviewed, and methods used.

Micsinszki et al (2022)	Peer-reviewed	Case study
Ministry of Children and Family Development BC (2013)	Non-peer reviewed	Report
Morris (2003)	Peer-reviewed	Case study
Moules (2012)	Peer-reviewed	Literature review
Nelson Ferguson et al (2023)	Peer-reviewed	Case study
Nesrallah et al (2023)	Peer-reviewed	Mixed-methods
Powell et al (2009)	Peer-reviewed	Qualitative study
Powers et al (2006)	Peer-reviewed	Literature review
Preston et al (2023)	Peer-reviewed	Commentary
Prior et al (2022)	Peer-reviewed	Qualitative study
Ross (2011)	Peer-reviewed	Case study
Salami et al (2021)	Peer-reviewed	Qualitative study
Scheve et al (2006)	Peer-reviewed	Qualitative study
Sexual Health and Reproductive Empowerment (2023)	Non-peer reviewed	Website
Sheikhan et al (2021)	Peer-reviewed	Qualitative study
Suleiman et al (2006)	Peer-reviewed	Case study
Viksveen et al (2022)	Peer-reviewed	Case study
Wadman et al (2019)	Peer-reviewed	Qualitative study
Walker et al (2021)	Peer-reviewed	Case study
Wilson et al (2007)	Peer-reviewed	Curriculum development
Woodgate et al (2018)	Peer-reviewed	Literature review
Woodgate (2021)	Non-peer reviewed	Website
